# Microfluidic Chamber Design for Organ-on-a-Chip: A Computational Fluid Dynamics Study of Pillar Geometry and Pulsatile Perfusion

**DOI:** 10.3390/bios16010049

**Published:** 2026-01-08

**Authors:** Andi Liao, Jiwen Xiong, Zhirong Tong, Lin Zhou, Jinlong Liu

**Affiliations:** 1 Institute of Pediatric Translational Medicine, Shanghai Children’s Medical Center, Shanghai Jiao Tong University School of Medicine, Shanghai 200127, China; andyliao777@sjtu.edu.cn (A.L.); xiongjiwen@scmc.com.cn (J.X.); tong-zhirong@scmc.com.cn (Z.T.); 2 Department of Cardiothoracic Surgery, Shanghai Children’s Medical Center, Shanghai Jiao Tong University School of Medicine, Shanghai 200127, China; 3 Shanghai Engineering Research Center of Virtual Reality of Structural Heart Disease, Shanghai Children’s Medical Center, Shanghai Jiao Tong University School of Medicine, Shanghai 200127, China; 4 State Key Laboratory of Transducer Technology, Shanghai Institute of Microsystem and Information Technology, Chinese Academy of Sciences, Shanghai 200050, China; zhoulinzlw@mail.sim.ac.cn

**Keywords:** organ-on-a-chip, microfluidic chamber, pillar geometry, pulsatile flow, computational fluid dynamics

## Abstract

Organ-on-a-Chip (OOC) platforms are microfluidic systems that recreate key features of human organ physiology in vitro via controlled perfusion. Fluid mechanical stimuli strongly influence cell morphology and function, making this important for cardiovascular OOC applications exposed to pulsatile blood flow. However, many existing OOC devices employ relatively simple chamber geometries and steady inflow assumptions, which may cause non-uniform shear exposure to cells, create stagnant regions with prolonged residence time, and overlook the specific effects of pulsatile perfusion. Here, we used computational fluid dynamics (CFD) to investigate how chamber geometry and inflow conditions shape the near-wall flow environment on a cell culture surface at a matched cycle-averaged volumetric flow rate. Numerical results demonstrated that pillarized chambers markedly reduced relative residence time (RRT) versus the flat chamber, and the small pillar configuration produced the most uniform time-averaged wall shear stress (TAWSS) distribution among the tested designs. Phase-resolved analysis further showed that wall shear stress varies with waveform phase, indicating that steady inflow may not capture features of pulsatile perfusion. These findings provide practical guidance for pillar geometries and perfusion conditions to create more controlled and physiologically relevant microenvironments in OOC platforms, thus improving the reliability of cell experimental readouts.

## 1. Introduction

Organ-on-a-Chip (OOC) technology is a cutting-edge approach that recreates key features of human organ physiology on a microfluidic cell culture chip by integrating engineering and biology [1]. It is widely regarded as a bridge between conventional cell culture and living organs. Static cell cultures struggle to reproduce the native microenvironment, dynamic mechanical cues and complex cell–cell interactions of human tissues, which limits the accuracy of predicted cellular responses to drugs and other stimuli [2,3]. In contrast, OOC models expose cells to controlled perfusion to reconstruct key aspects of organ level physiology in vitro [4]. As a result, OOC platforms provide powerful tools for drug testing and exploring disease mechanisms under near-physiological conditions, and hold promise for reducing reliance on animal models.

The cardiovascular system is particularly sensitive to hemodynamic forces that are absent in conventional static cell cultures. Hemodynamic flow is required for the development and maturation of the heart and vasculature, and its insufficiency can lead to malformation and functional abnormalities [5]. For example, zebrafish studies have shown that intracardiac flow dynamics regulate valve morphogenesis [6], and Alser et al. reported complementary findings in embryonic chick hearts, where altered hemodynamics resulted in a range of morphological defects [7]. These findings supported the critical role of flow that cannot be ignored in cardiovascular research models.

To recapitulate flow-dependent cues in vitro, OOC platforms perfuse culture medium through microfluidic chambers, generating controllable fluid shear stress along the cell surface. This provides an important biophysical stimulus that influences cell polarity, morphology and function. Several heart-on-a-chip studies have shown improved alignment and maturation in cardiomyocytes and endothelial cells under microfluidic perfusion [8,9]. In addition, continuous perfusion supplies fresh nutrients and oxygen while removing metabolic waste, supporting long-term culture of multilayered tissues that would otherwise undergo necrosis under static conditions [10].

Despite these advantages, achieving a controllable and uniform near-wall microenvironment remains a challenge. In many OOC systems, the flow field becomes unevenly distributed and may form stagnant zones near corners or junctions. This can lead to spatially heterogeneous shear exposure and uneven delivery of nutrients and oxygen to cells, ultimately compromising the consistency of cellular responses and experimental readouts [11]. Moreover, OOC perfusion is implemented using diverse driving methods, such as syringe, peristaltic, gravity-driven or pressure-controlled systems, and the resulting flow waveform can vary. It has been reported that pulsatile microfluidic flows may exert different effects on cells compared with steady perfusion, including nitric oxide production and morphological remodeling [12,13]. Together, these issues motivate a systematic evaluation of how chamber geometry and inflow patterns jointly shape the near-wall flow environment.

To support these design decisions, computational fluid dynamics (CFD) provides a practical approach by resolving detailed velocity fields, shear stress distributions and related metrics in complex microchannel geometries that are hard to measure or predict analytically. Accordingly, an increasing number of OOC studies have integrated CFD into the design process to guide channel layout and perfusion conditions [14,15,16]. For example, Bakuova N. et al. [14] conducted experiments using CFD to compare round and oval chamber geometries and evaluate filling dynamics, air entrapment and wall shear levels, demonstrating how device layout can be optimized before fabrication. More broadly, Pisapia et al. [16] compared four commonly used microchannel configurations and demonstrated how channel structure influences velocity and wall shear stress (WSS) distributions. Despite these advances, two gaps remain. First, steady inflow is still commonly assumed in OOC CFD studies, whereas perfusion driven by roller peristaltic pumps will introduce pulsatile waveforms that may alter near-wall flow development and shear exposure, which is particularly relevant for cardiovascular-oriented platforms. Second, the role of internal microstructures such as micropillars is less quantified. In particular, systematic parametric evaluation of pillar size on the spatial uniformity of shear over the cell culture surface remains limited.

In this study, we use CFD modeling to evaluate how internal pillar structures and inflow patterns shape the near-wall flow microenvironment in a perfused microfluidic OOC chamber. We compare a conventional flat chamber with two pillarized designs under both steady and peristaltic pulsatile perfusion, while matching the cycle-averaged volumetric flow rate across conditions. Fluid dynamic metrics on the cell culture surface are analyzed qualitatively and quantitatively, including time-averaged wall shear stress (TAWSS), Oscillatory Shear Index (OSI) and relative residence time (RRT). Our aim is to determine how internal pillars affect shear uniformity and near-wall residence, and how pulsatile perfusion further modulates these effects. The findings will provide design guidance for a more controllable and uniform flow environments in OOC systems, particularly cardiovascular-relevant applications.

## 2. Materials and Methods

### 2.1. Microfluidic Device Design

**Figure 1:**
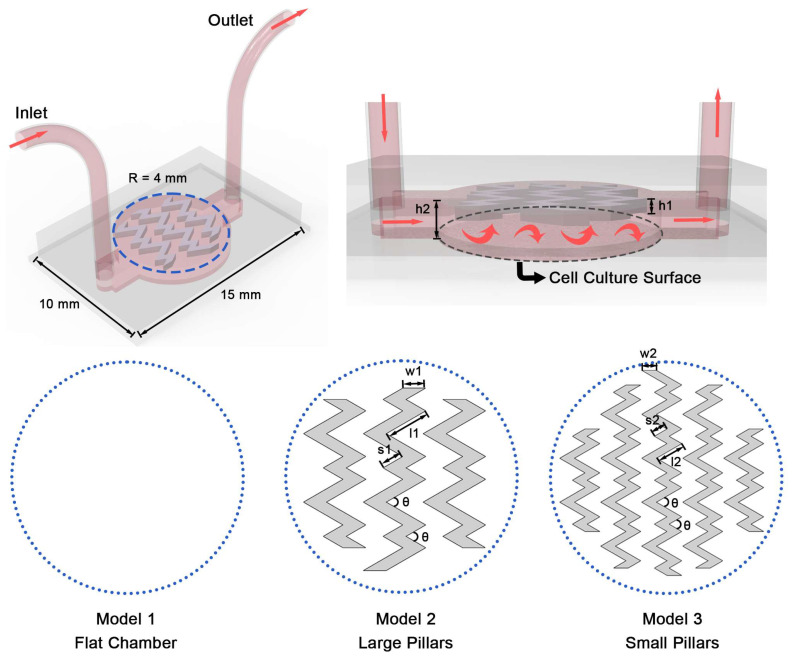
The overall structure and detailed dimensions of the OOC device. The blue dashed circles represent pillar structure, with a circular perfusion chamber of radius R = 4 mm and a fluid height of h1 = 1 mm. The pink arrows represent the expected flow direction. Model 1 features a flat chamber without pillars. In Model 2 and Model 3, the pillars have a height of h2 = 0.5 mm, and each herringbone pillar consists of a long segment (l) and a short segment (s) joined by a bend angle of θ = 60°. Model 2 incorporates larger herringbone pillars with l1 = 1.6 mm, s1 = 0.8 mm and w1 = 0.8 mm, while Model 3 incorporates smaller pillars with l2 = 1.0 mm, s2 = 0.5 mm and w2 = 0.5 mm.

The microfluidic OOC systems were designed in Solidworks 2021 (Dassault Systèmes SE, Vélizy-Villacoublay, France). The overall layout and detailed dimensions of Model 1, Model 2 and Model 3 are shown in Figure 1; all three models share the same principal dimensions and differ only in the presence and size of internal pillars. The blue dashed circle outlines the pillar region of the perfusion chamber, and the plan views show the pillar dimensions of each model. The gray dashed circle indicates the cell culture surface area, which was used as the analysis domain for subsequent hemodynamic calculations. A chamber height of 1 mm was selected to provide a stable perfusion microenvironment for cardiac tissue culture on the cell culture surface, consistent with published heart-on-a-chip studies that employ millimeter-scale channels [8,17].

Model 1 has a flat chamber without internal pillars and served as the control. Model 2 incorporates an array of larger herringbone pillars, whereas Model 3 incorporates an array of smaller pillars. The pillar height in both designs was fixed at 0.5 mm as a representative value to induce flow redistribution while avoiding excessive obstruction, and it was kept constant to isolate the influence of pillar geometry. The pillar arrangement was adapted from previously reported herringbone microchannel designs [18], in which turning pillar rows are used to enhance the mixing efficiency across the chamber.

### 2.2. CFD Analysis

#### 2.2.1. Governing Equations

The culture medium was modeled as an incompressible and Newtonian fluid with a density of 1020 kg/m^3^ and a viscosity of 0.0011 kg/(m s) [19], which could be solved via the following Navier–Stokes (NS) equations:

(1) $$\begin{array}{c} \left\{\begin{array}{c} \frac{\partial }{\partial t}\left(\rho u_{i}\right)+\frac{\partial }{\partial x_{j}}\left(\rho u_{i}u_{j}\right)=-\frac{\partial p}{\partial x_{i}}+\frac{\partial }{\partial x_{j}}\left[\mu \left(\frac{\partial u_{i}}{\partial x_{j}}+\frac{\partial u_{j}}{\partial x_{i}}\right)\right]+f_{i}\\ \frac{\partial \rho }{\partial t}+\frac{\partial }{\partial x_{j}}\left(\rho u_{j}\right)=0 \end{array}\right., \end{array}$$

where *u* is the velocity vector with components in the *x*, *y* and *z* directions, *ρ* is the fluid density, *μ* is the fluid viscosity, *p* is the pressure and *t* is the time. The term *f_i_* represents the action of body forces. Since the system is maintained at a constant temperature and the fluid is modeled as a single-phase liquid with constant density, buoyancy-driven convection is not expected. Under these conditions, gravity mainly contributes a hydrostatic pressure gradient, whereas the motion is continuously driven by the pump. Therefore, gravity was neglected in this study.

The Reynolds number (*Re*) was calculated to characterize the flow model used in the simulations:

(2) $$\begin{array}{c} Re=\frac{\rho UD}{\mu }, \end{array}$$

where *μ* is the medium viscosity, *ρ* is the medium density, *U* denotes the mean flow velocity and *D* denotes the characteristic length. In our study, the inlet diameter is 1 × 10^−3^ m; thus, the inlet *Re* was approximately 3.35, and even in regions of locally accelerated flow between pillars, the local *Re* remained below 20. These values were far below typical laminar–turbulent transition thresholds [20,21], similar to previous OOC studies where laminar flow is the most typical pattern implemented in microfluidic CFD models [22]. Therefore, a laminar flow model was adopted in our study.

#### 2.2.2. Mesh Generation

**Figure 2:**
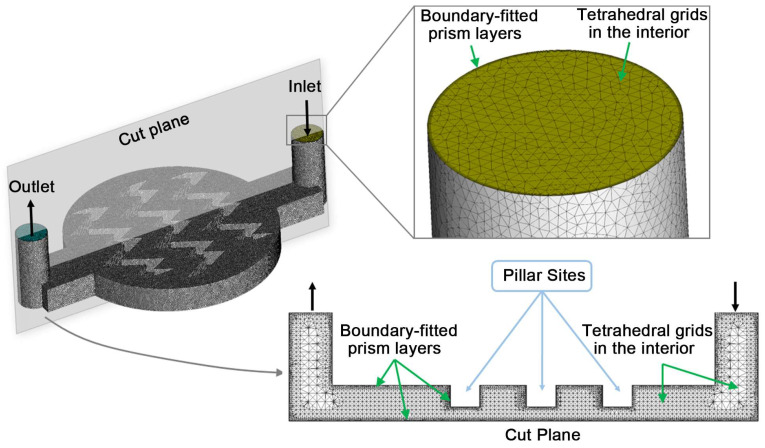
Schematic of the computational mesh.

Mixed grids for the simulations were generated with ANSYS^®^-ICEM CFD 2025 (ANSYS Inc., Canonsburg, PA, USA). Unstructured tetrahedral elements were used to fill the interior of the microfluidic chamber. To accurately resolve near-wall velocity gradients for WSS/TAWSS prediction, boundary-layer prism elements were generated on all fluid–solid interfaces, including the bottom culture surface, pillar faces and channel walls. Specifically, five prism layers were applied with a first-layer height of 2 μm and a growth rate of 1.2 (Figure 2). A mesh independence study was performed to identify an appropriate grid density. We compared progressively refined meshes and monitored the area-averaged TAWSS on the bottom culture surface as the primary indicator. When the total element count reached approximately 700,000, further refinement produced only a minor change in area-averaged TAWSS (<1.2%), indicating mesh independence. The meshes adopted for the final simulations are summarized in Table 1.

#### 2.2.3. Boundary Conditions

Transient simulations were performed for all inlet conditions, after obtaining steady solutions as initial fields where appropriate. Two inflow conditions were considered for each geometry: a pulsatile waveform and a steady inflow. In all cases, the target cycle-averaged volumetric flow rate was 170 μL/min. This flow rate was selected to achieve a gentle, experimentally achievable perfusion environment for cardiac culture on the chamber surface. Under this condition, the predicted shear on the culture surface remained mild, with TAWSS up to 0.035 Pa (0.35 dyn/cm^2^; 1 Pa = 10 dyn/cm^2^), comparable to shear levels reported for perfused engineered cardiac tissues [23,24]. A pressure outlet with zero pressure was imposed at the outlet for all models.

**Figure 3:**
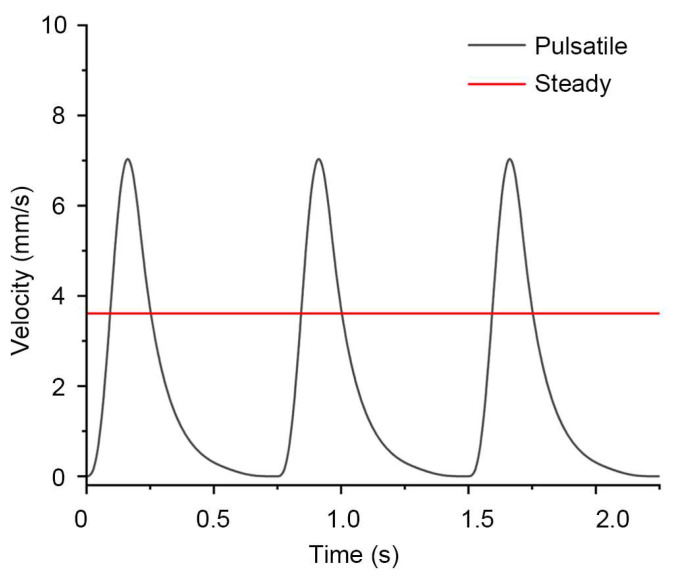
Inlet velocity waveforms for pulsatile and steady perfusion.

For the pulsatile inflow, we imposed a velocity waveform that mimics a roller peristaltic pump. Studies have shown that roller peristaltic pumps do not generate a continuous sinusoid; each roller pass creates a short pulse and the elastic tubing in the line partly smooths those pulses [25,26]. To capture this, we used a sequence of short, smooth raised-cosine pulses whose total area is set so that the time-average equals the targeted flow rate, as shown in Figure 3. Pulses repeat at a frequency calculated as below:

(3) $$\begin{array}{c} f=\frac{RPM}{60}\times R, \end{array}$$

where *RPM* is the pump speed and *R* is the number of rollers.

For the steady inflow, we applied an inlet velocity of 3.61 mm/s, corresponding to the same volumetric flow rate of 170 μL/min. To maintain a consistent post-processing procedure with the pulsatile simulations, we advanced a transient simulation with the same constant inlet velocity. The transient solver was run over the same physical time window as in the pulsatile simulations, that is, the time required for three pump cycles, to enable direct comparison between steady and pulsatile inflow conditions.

#### 2.2.4. Numerical Solution

The finite volume solver package ANSYS^®^-Fluent 2025 (ANSYS Inc., Canonsburg, PA, USA) was used to solve the transient incompressible flow in each model. All channel walls were treated as rigid surfaces with a no-slip boundary condition. The laminar flow model was applied. The semi-implicit method and the second-order upwind scheme were adopted to solve the discretized 3D incompressible NS equations. A steady-state solution with a constant inlet velocity was first obtained and used as the initial field for the subsequent transient simulations. A fixed time step of 1 × 10^−4^ s was used in all simulations, and convergence at each time step was assumed when all scaled residuals dropped below 10^−4^. Flow fields were saved every 20 time steps for post-processing.

#### 2.2.5. Calculation of Fluid Dynamic Metrics

WSS characterizes the interaction between the culture medium and the wall of the microfluidic chamber. In the simulations, the wall shear stress vector was obtained directly from Fluent, and its magnitude *τ_w_* was used for subsequent analyses.

TAWSS indicates the time-averaged WSS. It can be calculated via the following equation:

(4) $$\begin{array}{c} \mathrm{TAWSS}=\;\frac{1}{T}\int_{0}^{T}\left|\tau_{w}\right|dt, \end{array}$$

where *τ_w_* is the instantaneous wall shear stress magnitude and *T* is the duration of a perfusion cycle.

OSI is a dimensionless measure of the degree of oscillation in the wall shear stress direction during a perfusion cycle, where OSI = 0 corresponds to purely unidirectional shear and OSI = 0.5 indicates a fully oscillatory shear with zero net direction [27]. It can be calculated via the following equation:

(5) $$\begin{array}{c} \mathrm{OSI}=\;\frac{1}{2}\left[1-\frac{|\int_{0}^{T}\tau_{w}dt|}{\int_{0}^{T}|\tau_{w}|dt}\right], \end{array}$$

RRT, originally introduced by Himburg et al., integrates the effects of TAWSS and OSI into a near-wall residence metric that reflects how long fluid elements remain close to the wall [28]. It can be calculated via the following equation:

(6) $$\begin{array}{c} \mathrm{RRT}\;\sim \;\frac{1}{\left(1-2\times OSI\right)\times TAWSS}, \end{array}$$

To quantify the spatial uniformity of TAWSS on the cell culture surface, we defined a TAWSS uniformity index based on the mean and standard deviation of TAWSS.

The mean TAWSS was calculated as

(7) $$\begin{array}{c} \mu =\frac{1}{N}\sum_{i=1}^{N}\tau_{i}, \end{array}$$

where *N* is the number of computational cells into which the cell culture surface region of interest was discretized, and *τ_i_* denotes the local TAWSS at cell *i*.

The standard deviation was calculated as

(8) $$\begin{array}{c} \sigma =\sqrt{\frac{1}{N}\sum_{i=1}^{N}{\left(\tau_{i}-\mu \right)}^{2}}, \end{array}$$

The coefficient of variation was

(9) $$\begin{array}{c} CV=\frac{\sigma }{\mu }, \end{array}$$

and the uniformity index was defined as

(10) $$\begin{array}{c} U\%=\left(1-CV\right)\times 100, \end{array}$$

such that *U%* approaches 100% for spatially uniform TAWSS distributions and decreases as TAWSS becomes more heterogeneous.

## 3. Results

### 3.1. Relative Residence Time

**Figure 4:**
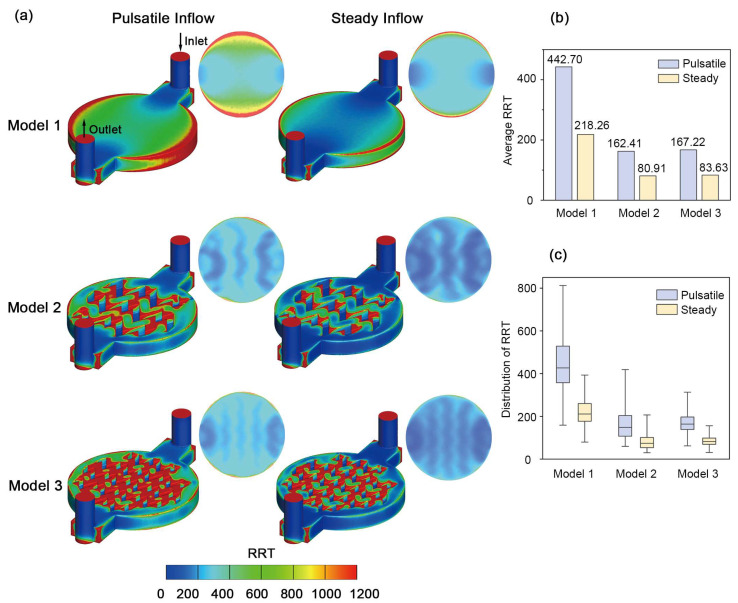
RRT maps and distributions for the three models under pulsatile and steady inflow. (**a**) RRT colormaps for the three geometries under pulsatile and steady inflow; each combination includes an overall view of the chip and the extracted bottom cell culture surface; flow is from inlet to outlet as indicated in the upper left panel, and the same orientation applies to all subpanels; a common color scale is used for all panels. (**b**) Bar charts of average RRT on the cell culture surface for each condition. (**c**) Box plots of RRT, showing central tendency and spread. The box represents the 25–75th percentiles, and the median is indicated. The whiskers show the range of values.

As shown in Figure 4, RRT patterns differed markedly across chamber geometries and inflow conditions. From the overall view of the chip in Figure 4a, high-RRT regions appeared along the outer edges of the chamber in Model 1, while in Model 2 and Model 3, high-RRT regions were concentrated near the corners of the pillars. The bottom surface of each chip, corresponding to the cell culture surface, is shown separately next to the overall view of the chip. Focusing on the cell culture surface, Model 1 exhibited pronounced high-RRT regions along the chamber borders, while the pillarized chambers in Model 2 and Model 3 showed few high-RRT regions both around the borders and in the central area. Mechanistically, slow flow near the borders in Model 1 made the periphery more prone to prolonged residence. In contrast, the pillar arrays redistribute the flow and help break up the continuous low-velocity region along the chamber edges, thereby improving clearance. Notably, regions beneath the pillars showed lower RRT than adjacent areas, possibly caused by higher local velocity and shear at the relatively narrow passages under the pillars, whereas the high-RRT spots around the pillar corners may have resulted from locally sheltered zones created by the pillars. Comparing the pulsatile and steady inflow, all geometries exhibited fewer high-RRT regions under steady inflow compared to pulsatile inflow, indicating that pulsatile inflow tended to increase fluid residence times on the cell culture surface.

These spatial trends are reflected quantitatively in the bar chart in Figure 4b, which shows the average RRT values on the cell culture surface for each geometry and inflow condition. Across the three geometries, Model 1 exhibited the highest RRT values, while a pronounced decrease was exhibited in the Model 2 and Model 3 counterparts in both pulsatile and steady inflow. However, the values of Model 2 and Model 3 were similar and showed no clear differences, suggesting that the two pillar designs had comparable effects on average RRT. All values in pulsatile perfusion were statistically higher than steady perfusion and alterations to inflow conditions did not change the ranking of three models, which was consistent with the colormap observations described above. Notably, in both Model 2 and Model 3, RRT values under pulsatile inflow were lower than those in Model 1 under steady inflow, suggesting that adding pillars to the chamber had a stronger effect on RRT than switching between pulsatile and steady perfusion.

We further analyzed differences in RRT variability between models, as shown in Figure 4c. Although the mean RRT values of Model 2 and Model 3 were similar, the variations in Model 3 were clearly lower. Model 3 exhibited the smallest interquartile range and the tightest spread, indicating more uniform RRT across the cell culture surface. Compared with Model 1, both pillarized chambers reduced the variability of RRT. These results suggested that pillarized chamber could contribute to both lower average RRT values and less variations in RRT distributions on the cell culture surface. Specifically, Model 3 with small pillars suppressed extreme RRT values more effectively and yielded a tighter distribution than Model 2 with large pillars. The tighter RRT distribution in Model 3 may be explained by the smaller pillars, which tend to break up stagnation into smaller, weaker pockets. As a result, extreme RRT values are suppressed and the overall spread becomes narrower. These trends held the same under both pulsatile and steady perfusion. Taken together, these data showed that introducing pillars into OOC platforms, particularly smaller pillars, helps to reduce fluid stagnation and suppress extreme RRT that could lead to non-uniform exposure of cells.

### 3.2. Velocity Magnitude and Oscillatory Shear Index

**Figure 5:**
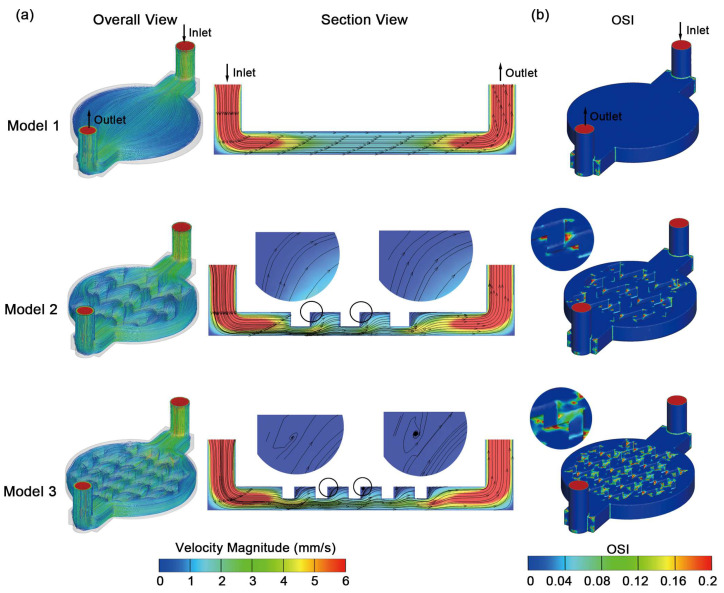
Flow organization and OSI under pulsatile perfusion. Flow is from inlet to outlet as indicated in the upper row, and the same orientation applies to all subpanels. (**a**) Velocity streamlines at peak inflow velocity, shown as an overall chip view and a longitudinal mid-plane section for each geometry. Magnified views highlighted by black circles are shown above each sectional image. A common color scale is used for all velocity panels. (**b**) OSI colormaps for each geometry; a common color scale is used for all OSI panels.

Figure 5a shows the overall and sectional views of the velocity streamline patterns at the peak of the pulsatile cycle, and Figure 5b shows overall OSI colormaps. From the overall chip view, it can be seen that the velocity streamlines closely followed the chamber contours. Then, we extracted the longitudinal mid-plane section to observe more detailed flow features. In Model 3 with small pillars, the sectional view revealed confined eddies at the inner turning corners of pillars, whereas Model 2 with larger pillars did not show such clearly defined corner eddies. Our further observations of OSI distributions were consistent with these velocity patterns, where relatively high-OSI regions co-localized with the pillar corners where the flow was most disturbed. Both pillarized chambers in Model 2 and Model 3 exhibited relatively high-OSI regions around the turning corner of pillars, indicating that pillarized design introduced directional oscillation of near-wall shear. Notably, Model 2 with large pillars showed more localized peak-OSI regions, whereas Model 3 with small pillars presented a broader area with moderately elevated OSI.

It is important to mention that all models exhibited very low OSI values around 7.4 × 10^−4^ to 9.4 × 10^−4^ on the cell culture surface (Table 2), indicating negligible differences across models and inflow conditions. This is likely because the pillars are located in the upper layer of the chamber where they introduce local directional fluctuations, whereas the bottom surface was separated from these disturbances and remained dominated by a largely unidirectional near-wall flow. Notably, the low OSI does not indicate an absence of pulsatility. Instead, the shear stress magnitude underwent pronounced cyclic variation, while its primary direction remained stable on the culture surface. Therefore, directional oscillation at the cell culture layer was minimal, so it was reasonable to assume that the RRT behavior reported in Figure 4 was driven primarily by geometry and shear stress magnitude, rather than strong bidirectional shear at the cell culture surface. In addition, it also suggested that high-OSI regions in the upper layer of the chamber did not propagate down to the bottom surface, limiting the exposure of cells to unfavorable oscillatory shear.

### 3.3. Time-Averaged Wall Shear Stress

**Figure 6:**
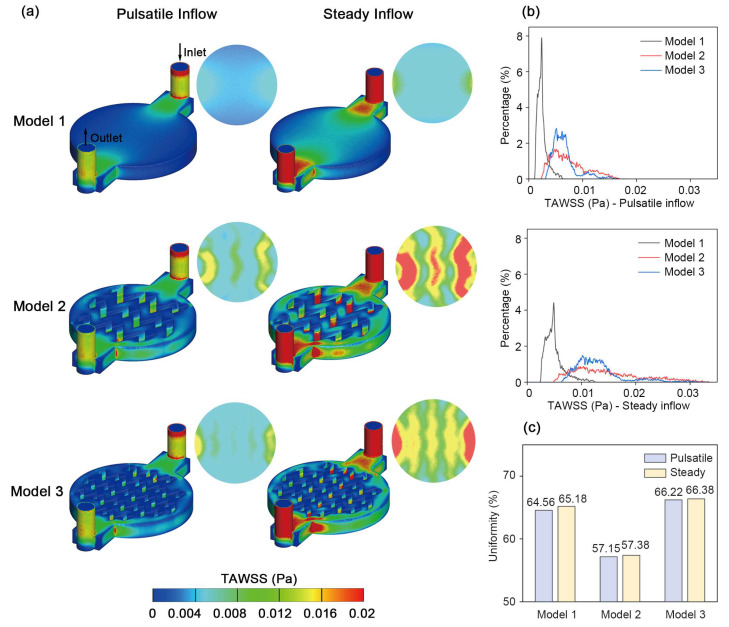
TAWSS maps and distributions for the three models under pulsatile and steady inflow. (**a**) TAWSS colormaps for the three geometries under pulsatile and steady inflow; each combination includes an overall view of the chip and the extracted bottom cell culture surface; flow is from inlet to outlet as indicated in the upper left panel, and the same orientation applies to all subpanels; a common color scale is used for all panels. (**b**) TAWSS distribution curves on the culture surface under pulsatile (up) and steady (down) inflow. (**c**) TAWSS uniformity index for each model and inflow condition.

Figure 6 presents the TAWSS maps and distributions, illustrating the time-averaged shear landscapes for different geometries and flow conditions. Compared with the flat chamber in Model 1, which showed a low overall value on the cell culture surface, the pillarized chambers in Model 2 and Model 3 exhibited locally elevated TAWSS beneath the pillars, in line with the low RRT observed in these regions described in Figure 4. This pattern is expected because the flow is locally squeezed through narrower passages, increasing near-wall velocity gradients and therefore shear stress. The same accelerated pathways also promote convective washout, explaining the co-location of higher TAWSS and lower RRT beneath the pillars. This effect was more prominent under steady inflow than pulsatile inflow, as shown in Figure 6a.

The corresponding distributions of TAWSS values are presented in Figure 6b. Model 1 exhibited the narrowest absolute TAWSS range and a sharp peak in the histogram. In contrast, the pillarized chambers in Model 2 and Model 3 elevated overall values and broadened the TAWSS range. Specifically, Model 2 with large pillars presented the widest range and the most dispersed distribution. Comparing the pulsatile and steady inflow conditions, steady inflow yielded higher TAWSS magnitudes, a broader range and more dispersed distributions than pulsatile inflow for all geometries, consistent with the qualitative patterns in the colormaps.

To compare shear homogeneity more directly, we evaluated the TAWSS uniformity index for each model and inflow condition, as shown in Figure 6c. The results complemented the former distribution observations: Model 3 with smaller pillars achieved the highest TAWSS uniformity, slightly exceeding Model 1 with a flat chamber, while Model 2 held the lowest uniformity. Differences in uniformity between pulsatile and steady perfusion were modest for all geometries.

### 3.4. Wall Shear Stress Comparison Between Matched Pulsatile and Steady Inflow

**Figure 7:**
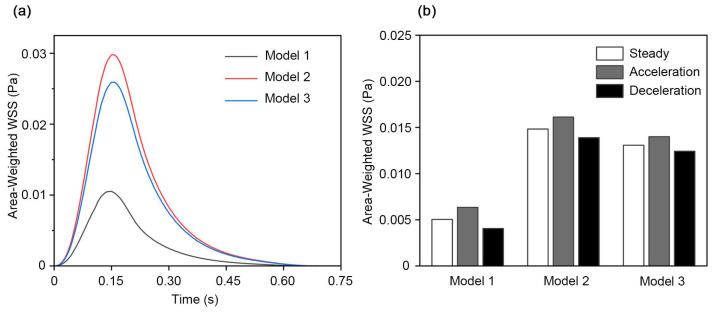
WSS over a full pulsatile cycle and at a matched inflow velocity across different flow phases. (**a**) Area-weighted WSS on the culture surface of the three models over one pulsatile cycle of perfusion. (**b**) Comparison of area-weighted WSS for each model at an inflow velocity of 3.61 mm/s during the pulsatile acceleration phase, steady inflow, and pulsatile deceleration phase.

We compared the area-weighted wall shear stress (WSS) over a full pulsatile cycle for the three models. Figure 7a shows the area-weighted WSS across one pulsatile cycle, which followed a stable ranking: Model 2 exhibited the highest values, Model 3 was slightly lower, while Model 1 showed the lowest values and was clearly below the other two models.

Then, we extracted the area-weighted WSS values at time points with the same instantaneous inflow velocity to investigate differences at a matched inflow speed. Figure 7b compares the area-weighted WSS for each model at the same inflow velocity of 3.61 mm/s. For all three models, the pulsatile acceleration phase exhibited the highest WSS, the deceleration phase showed the lowest, and steady inflow gave intermediate values. The results suggested that even at an identical inflow velocity, WSS depended not only on geometry but also the flow phase. This phase effect is expected in unsteady flows, because acceleration and deceleration do not produce identical near-wall conditions. The waveform can induce phase-dependent flow redistribution above the culture surface and lead to different near-wall velocity profiles, so the near-wall gradient may be steeper during acceleration and weaker during deceleration. Therefore, using a steady inflow in OOC platforms could neglect changes in WSS over the cycle and lead to misestimation of the temporal variability of WSS.

### 3.5. Summary

Compared with Model 1 (the flat chamber), both Models 2 and 3 (large and small pillarized chambers) reduced mean RRT on the cell culture surface, with Model 3 additionally showing a more uniform RRT distribution. TAWSS was generally higher in Models 2 and 3 than in Model 1. Among pillarized designs, Model 3 provided the most uniform TAWSS distribution, whereas Model 2 exhibited reduced TAWSS uniformity. OSI on the culture surface remained very small across all models and inflow conditions, with negligible differences. Pulsatile perfusion increased RRT and reduced TAWSS compared with steady perfusion at matched mean flow rate. At the same instantaneous flow rate, WSS differed between pulsatile and steady conditions, indicating phase-dependent behavior. A schematic linking CFD metrics to their biological interpretation is provided in Figure S1.

## 4. Discussion

This study investigated how chamber geometry and inflow conditions shape the fluid dynamic environment in an OOC platform, particularly at the cell culture surface. We identified three major findings: First, pillarized chambers reduced near-wall stagnation and improved clearance compared with a flat chamber, as reflected by lower RRT. In particular, Model 3 with small pillars produced more uniform RRT and TAWSS distributions than both the flat chamber and the large pillar design, illustrating the importance of rational pillar arrangement and providing a practical pillar configuration for OOC platforms. Second, regions of high oscillatory shear generated around the pillars in the upper layer of the chamber did not propagate down to the culture surface, demonstrating that this pillar design can be used to improve uniformity and clearance while keeping the cell layer protected from unfavorable oscillatory shear; thus, it is a feasible design strategy for OOC platforms. Third, physiological pulsatile perfusion cannot simply be replaced by steady perfusion. Our results suggested that WSS depended not only on geometry but also the flow phase; thus, using steady perfusion in OOC platforms may overlook changes in WSS over the cycle and associated cellular responses.

First, we evaluated RRT as an integrated indicator of near-wall clearance. RRT is widely interpreted as a near-wall stagnation metric in cardiovascular CFD studies, used to estimate the relative amount of time particles or cells may be in contact with the blood vessel wall [29,30], and it has also been suggested that RRT is associated with drug transport and efficacy [31]. However, RRT does not have an absolute threshold value for adequate nutrient delivery or waste removal. In our study, we assumed that high RRT may promote local depletion of nutrients and accumulation of waste, which was also consistent with the results of microfluidic cell culture research by Gu et al., who demonstrated a gradient of reduced cell growth and density downstream of perfusion, presumably due to consumption of nutrients and oxygen and buildup of waste in the long-residence fluid [32].

In our study, both pillarized models showed lower average RRT values on the cell culture surface, indicating more effective clearance than the conventional flat chamber. This trend is consistent with experimentally assessed pillarized OOC systems, where adding pillar arrays accelerated dye washout via pillar-induced changes in fluid distribution and improved exchange performance under perfusion [33,34]. Although the pillar geometries and operating conditions differ across platforms, these studies support the general mechanism that pillar arrays reshape perfusion and clearance.

Moreover, our results showed that Model 3 with smaller pillars further reduced the spread of RRT distributions, providing cells with a more spatially uniform residence time distribution on the culture surface. More broadly, high RRT is usually associated with negative cellular outcomes. For example, D. Lee et al. suggested in their vascular-on-a-chip research that higher RRT retained particles and potentially increased pathological endothelial–blood cell interactions [35]. Another study by Tzirakis et al. showed that RRT has a positive association with thrombus growth [36], and Trenti et al. found in their study that endothelial cells in high-RRT regions took up more inflammatory molecules, with increased oxidative stress and permeability [37]. Thus, it is reasonable to assume that extremely high-RRT regions around the borders of conventional flat chamber of Model 1 could lead to spatially non-uniform nutrient and waste exchange and potentially adverse cell responses, while pillarized models, especially Model 3 with small pillars, provide a more uniform medium renewal across the culture surface, which may enhance the reliability of OOC platforms. In practice, cells are often introduced into the closed chamber by injection through inlet ports, so cell coverage near the periphery is difficult to completely avoid. Although imaging readouts such as immunofluorescence often focus on a central region, regions of high RRT near the boundaries may still influence assays that integrate signals across the whole chip, such as ELISA or qPCR, and thus introduce additional variability or bias. This further highlights the importance of optimizing chamber design. We also suggest using a consistent central region for analysis when possible. If feasible, seeding more toward the center may help minimize peripheral effects.

Beyond clearance, another design objective is shear homogenization on the culture surface, which we quantify using the TAWSS distribution and its uniformity index. The advantages of Model 3 with small pillars were also shown in the TAWSS distributions, exhibiting the highest uniformity and controllability. In a previous CFD study, Kim et al. used micropillar arrays to generate multiple shear-stress levels across parallel regions, supporting the idea that micropillar arrays could provide a practical approach to adjust the average shear and shape its spatial distribution across the culture area [38]. Our results indicated that cells across the culture surface in Model 3 would experience more similar shear levels. Such homogenization of shear exposure is beneficial for long-term perfusion cultures, because it makes the mechanical stimulus more predictable and easier to control. Prior studies have shown that large spatial variations in WSS can drive heterogeneous endothelial signaling and cellular responses [39,40], whereas more uniform laminar shear tends to promote stable phenotypes, characterized by upregulation of KLF2 and eNOS and suppression of inflammatory and thrombotic genes [41,42,43]. As a result, a more uniform and controllable TAWSS distribution is desirable for precise control and obtaining consistent readouts.

OSI distributions further support the feasibility of the pillarized OOC chamber. Following previous CFD and clinical hemodynamic studies, regions with OSI over 0.2 are usually classified as disturbed flow zones associated with adverse endothelial responses such as remodeling, inflammation and endothelial dysfunction [44,45,46]. Therefore, in our OOC platform we interpreted increases in OSI as a marker of unfavorable oscillatory shear exposure. While we found high-OSI regions around the corners of pillars on the upper layer, the average OSI on the cell culture surface remained extremely low and showed minor differences between models. These results indicated that the effects of pillars on OSI were limited to the upper layer of the chamber without introducing unfavorable oscillatory shear exposure to cells, further supporting the rationality of this pillar design. Notably, pulsatility was still present and was manifested primarily as cyclic variation in WSS magnitude, as reflected by TAWSS and RRT. Thus, the pillars improved clearance (reduced RRT) and promoted a more uniform shear distribution (improved TAWSS uniformity) while keeping directional oscillation at the culture surface minimal, thereby limiting exposure to potentially unfavorable bidirectional oscillatory shear.

Finally, our comparisons of WSS between matched inflow conditions highlighted that instantaneous wall shear was determined not only by geometries and instantaneous inflow velocity, but also the flow phases. Our results showed that at identical inflow velocity, the WSS of pulsatile acceleration exceeded that of the steady case, and the steady case exceeded that of pulsatile deceleration. To date, many OOC CFD studies have simplified perfusion as steady inflow, while practical pumping systems such as peristaltic pumps can introduce pulsatility. Similar limitations of steady inflow have also been reported by Wain et al. in their hemodynamic CFD study [47]. They showed that using a steady inflow simulation driven by the peak systolic velocity significantly underestimated the maximum shear strain rate compared with realistic pulsatile flow, highlighting that unsteady effects can amplify peak shear even when instantaneous velocities are matched. This is consistent with our observations and further supports the use of pulsatile inflow to capture shear features that are missed by steady approximations. For cardiovascular-related research, pulsatile flow is a defining feature of the hemodynamic environment. Hsiai et al. found marked differences in the impact of pulsatile flow on cells compared to steady flow, with significantly increased rates of endothelial cell elongation and realignment [12]. It was also found by Yee et al. that pulsatile shear stress significantly decreased NO production in HUVEC relative to steady shear stress [13]. All these studies, including our findings, indicated that physiological pulsatile inflow cannot be simply replaced by steady inflow in OOC platforms.

This study has several limitations. First, we investigated a single representative peristaltic pump-induced waveform and only one representative flow rate was evaluated. The waveform captures the pulsatility commonly introduced by roller pumps and did not reproduce the full range of physiological cardiovascular waveforms. Future work will explore a wider range of flow rates and perform a systematic analysis across waveform parameters, such as frequency and peak-to-mean ratio. Second, the geometric parameter space was explored only partially. We tested a limited set of pillar types and sizes within a fixed chip size and chamber height, while flow characteristics may vary with pillar shape, arrangement and chamber height. Applying the present design conclusions to other channel designs and dimensions would require additional optimization and will be addressed in future work, with the goal of establishing more general design criteria for shear homogeneity. Third, the present work focused on flow characterization and structural optimization and did not yet include cell or organoid layers. While a cellular layer may slightly alter the effective boundary condition and local shear, its thickness is low relative to the chamber height in our platform, so the impact is expected to be small and unlikely to affect the overall conclusions. Moreover, realistic cell morphology is heterogeneous and time-varying, making it difficult to represent explicitly in CFD. Future work may further evaluate these effects and use endothelial and cardiomyocyte cultures to assess biological relevance. Fourth, we modeled the chamber wall as rigid, while PDMS is compliant. In our simulations, the inlet–outlet total pressure drop remained very low (<5 Pa), suggesting that wall deformation would be negligible and unlikely to materially affect the reported trends. Future work may incorporate fluid–structure interaction modeling to quantify the impact of PDMS compliance under more demanding loading conditions.

## 5. Conclusions

In conclusion, our study showed that chamber geometry and inflow conditions markedly shaped the near-wall fluid dynamic environment in perfused OOC platforms. Introducing pillar arrays into a perfused chamber improved more effective clearance, as reflected by reduced RRT, and smaller pillars further promoted more uniform shear exposure on the cell culture surface among the tested geometries. Although localized high OSI appeared near pillar corners in the upper layer of the chambers, OSI remained very low at the cell culture surface, supporting the rationale for the current pillar design. Moreover, time-resolved analysis indicated that pulsatile flow cannot be reliably replaced by steady perfusion even at matched instantaneous flow rates, because WSS depends on flow phase under unsteady conditions and thus may better reflect the shear stimulus experienced by cells. These findings provide practical guidance for designing cardiovascular-relevant OOC systems and encourage future experimental validation to link the improved fluid dynamic metrics to biological outcomes.

## Figures and Tables

**Table 1:** Mesh information of each model.

	Model 1	Model 2	Model 3
Total elements	715,261	773,756	822,122
Total nodes	127,223	138,175	146,276

**Table 2:** Average OSI on the cell culture surface for the three models under pulsatile and steady inflow.

Geometry	Pulsatile Inflow (×10^−4^)	Steady Inflow (×10^−4^)
Model 1	9.40	8.23
Model 2	8.70	8.24
Model 3	7.91	7.46

## Data Availability

The original contributions presented in this study are included in the article/Supplementary Material. Further inquiries can be directed to the corresponding author.

## Supplementary Materials

The following supporting information can be downloaded at: https://www.mdpi.com/article/10.3390/bios16010049/s1, Figure S1: Conceptual framework connecting CFD metrics to biological interpretation in the OOC chamber.

## Abbreviations

The following abbreviations are used in this manuscript:

CAD	Computer-aided Design
CFD	Computational Fluid Dynamics
NS	Navier–Stokes
OOC	Organ-on-a-Chip
OSI	Oscillatory Shear Index
RRT	Relative Residence Time
TAWSS	Time-Averaged Wall Shear Stress
WSS	Wall Shear Stress
