# Supplementary figures and images for: Microfluidic Chamber Design for Organ-on-a-Chip: A Computational Fluid Dynamics Study of Pillar Geometry and Pulsatile Perfusion
Source: Biosensors (Basel). 2026 Jan 8;16(1):49. doi: 10.3390/bios16010049 (PMC12839026; doi:10.3390/bios16010049)

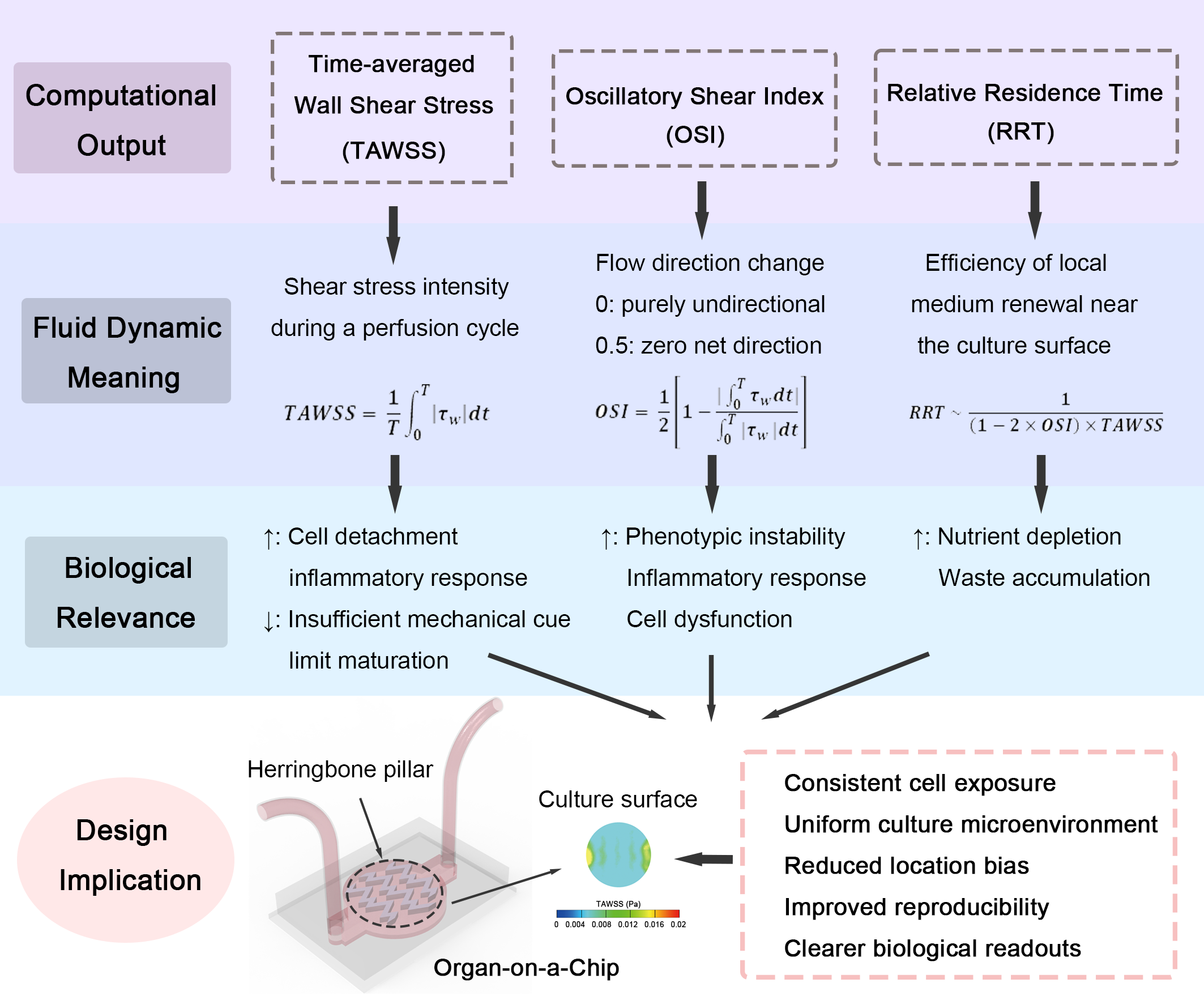

Supplement: Supplementary file 1 [file biosensors-16-00049-s001.zip › Supplementary Figure S1.tif]
